# Supplementary material for: The interactome of intact mitochondria by cross-linking mass spectrometry provides evidence for coexisting respiratory supercomplexes
Source: Mol Cell Proteomics. 2017 Dec 8;17(2):216–32. doi: 10.1074/mcp.RA117.000470 (PMC5795388; doi:10.1074/mcp.RA117.000470)
Supplement: Supplemental Data [file supp_RA117.000470_133922_0_supp_23978_fzffwf.zip › spectra_annotation/mito_DR_spectra_annotation/376-1-11-1-7-1.pdf]

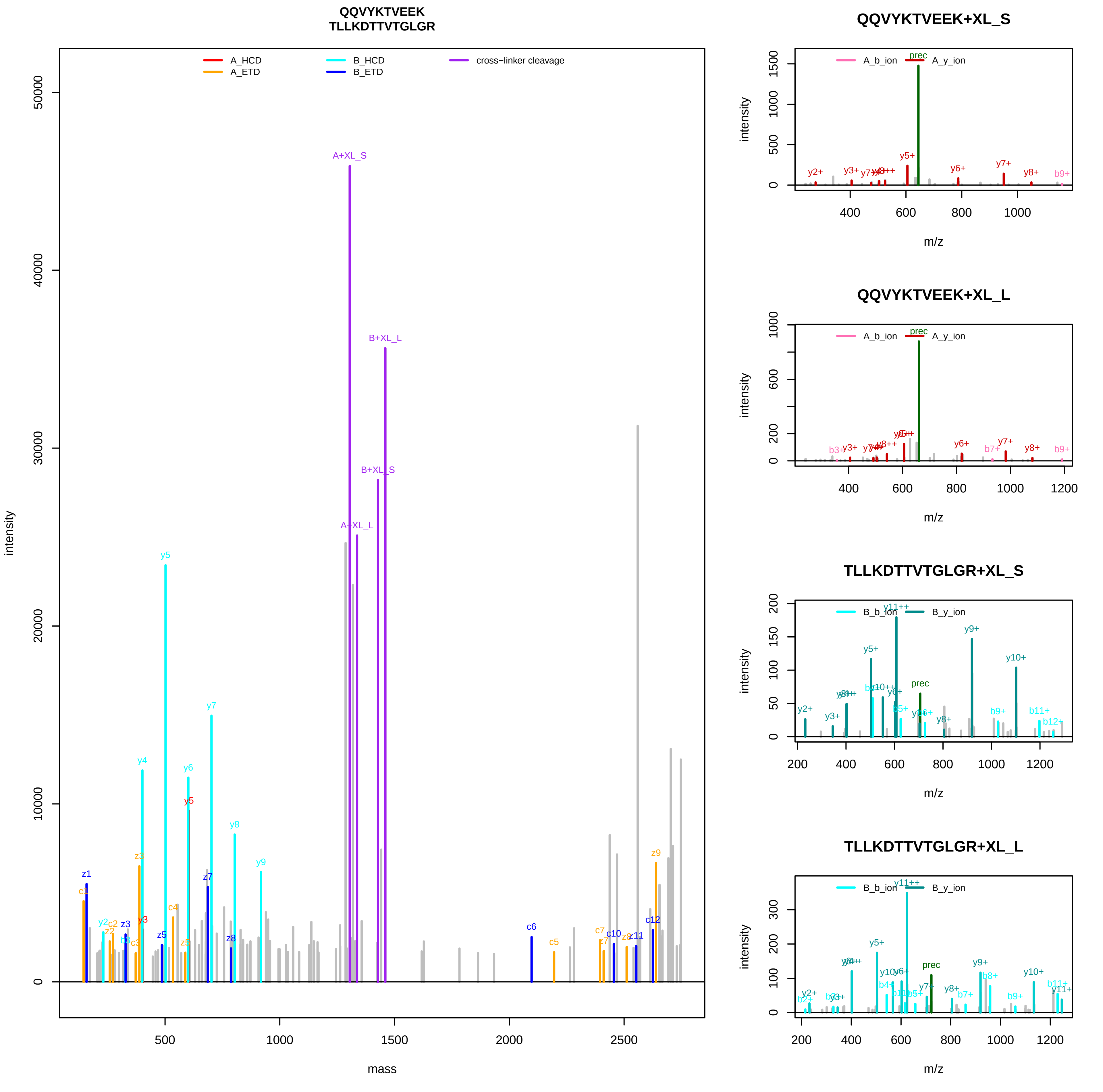
QQVYKTVEEK
TLLKDTTVTGLGR
A_HCD
A_ETD
B_HCD
B_ETD
cross−linker cleavage
intensity
mass
0
10000
20000
30000
40000
50000
500
1000
1500
2000
2500
A+XL_S
B+XL_L
B+XL_S
A+XL_L
y5
y7
y4
y6
y5
y8
y9
z1
c1
y2
c2
z2
z3
z3
y3
c3
c4
z5
z5
z7
z8
c6
c5
c7
z7
c10
z11
c12
z9
QQVYKTVEEK+XL_S
A_b_ion
A_y_ion
prec
intensity
m/z
0
500
1000
1500
400
600
800
1000
y2+
y3+
y5+
y6+
y7+
y8+
b9+
QQVYKTVEEK+XL_L
A_b_ion
A_y_ion
prec
intensity
m/z
0
200
600
1000
400
600
800
1000
1200
b3+
y3+
y6+
b7+
y7+
y8+
b9+
TLLKDTTVTGLGR+XL_S
B_b_ion
B_y_ion
intensity
m/z
0
50
100
150
200
200
400
600
800
1000
1200
y11++
y9+
y5+
y10+
prec
y2+
y3+
y6+
b5+
y8+
b9+
b11+
b12+
TLLKDTTVTGLGR+XL_L
B_b_ion
B_y_ion
intensity
m/z
0
100
200
300
200
400
600
800
1000
1200
y11++
y5+
prec
y9+
y10+
b8+
y8+
b7+
b9+
y11+
b2+
y2+
b4+
